# Nurses’ Perspectives on Evidence Dissemination Barriers and Large Language Model–Based Support: Qualitative Study Using Focus Groups and Nominal Group Technique

**DOI:** 10.2196/80289

**Published:** 2025-11-07

**Authors:** Junyi Ruan, Yimin Tang, Zhongyu Wei, Weijie Xing, Yan Hu

**Affiliations:** 1 School of Nursing Fudan University Shanghai China; 2 School of Data Science Fudan University Shanghai China; 3 Shanghai Innovation Institute Shanghai China; 4 JBI Fudan University Centre for Evidence-Based Nursing Shanghai China

**Keywords:** evidence dissemination, evidence-based nursing, focus group, large language models, nominal group technique

## Abstract

**Background:**

Current evidence dissemination methods fall short of meeting clinical nurses’ needs, hindering the implementation of evidence-based nursing practice. Large language models (LLMs), with their advanced natural language processing capabilities, offer potential as innovative tools to facilitate evidence dissemination. However, general-purpose LLMs typically lack domain-specific knowledge, are insufficient to support effective evidence dissemination in clinical contexts. It is essential to develop artificial intelligence tools tailored to nurses’ needs and preferences to enhance evidence dissemination.

**Objective:**

The aim of this study is to identify the challenges and barriers clinical nurses face in disseminating evidence, examine their perspectives on the use of existing LLMs to support evidence dissemination, and explore their needs and preferences regarding an LLM-based nursing evidence question-answering system.

**Methods:**

This qualitative study used a combined method of focus group discussions and the nominal group technique (NGT). Using purposive sampling, nurses with diverse specialties, professional titles, and years of experience were recruited, resulting in a total of 22 clinical nurses who completed the entire study. A total of 2 focus group discussions were conducted online via Tencent Meeting between November and December 2024 to explore the challenges and barriers nurses face in disseminating evidence, as well as their perspectives on using existing LLMs to support evidence dissemination. The data were analyzed using qualitative content analysis following the approach of Graneheim and Lundman. Subsequently, the NGT was used between March and April 2025 to identify nurses’ needs and preferences for the system to be developed. To overcome geographical constraints and participants’ busy schedules, the NGT was conducted entirely online, using online questionnaires and WeChat groups. Overall, 2 rounds of voting were conducted to determine the priority ranking of the functionalities.

**Results:**

The focus group yielded 3 main themes and 7 subthemes. Three main themes were identified as (1) pathways for evidence dissemination among nurses, (2) barriers that hinder the effective dissemination of evidence, and (3) advantages and limitations of using LLMs to support evidence dissemination. The limitations of current LLMs served as the foundation for nurses’ subsequent reflections in the nominal group discussions on the desired functions of a newly developed LLM. The NGT sessions ultimately identified 9 desired functions. After prioritization, the top 3 ranked functions were evidence-based, high-quality question-answering, evidence source provision, and personalized evidence recommendation.

**Conclusions:**

The current evidence dissemination process faces multiple barriers. LLMs hold promise as innovative tools to support evidence dissemination, but require further refinement. Clinical nurses have identified key functional needs, guiding the development of LLMs specifically tailored to clinical nursing practice.

## Introduction

Although evidence-based practice has been shown to improve care quality, patient outcomes, and health system efficiency [1], its integration into clinical settings remains suboptimal. In China, many clinical nurses have limited opportunities for advanced education and specialized training in evidence-based knowledge, which has been identified as an important barrier to evidence implementation [2]. Additionally, constrained time, heavy workloads, and insufficient expert guidance have been reported to hinder nurses’ access to and application of evidence [3,4]. Moreover, although academic institutions are actively engaged in promoting evidence dissemination, current pathways remain insufficiently effective, delaying the uptake of research findings and limiting clinical nurses’ awareness of emerging evidence [5]. Therefore, to advance the implementation of evidence-based nursing practice, it is essential to strengthen evidence dissemination and support nurses’ use of the best available evidence [6].

Evidence dissemination refers to the active and targeted distribution of research findings to promote their application in practice. Evidence dissemination plays a pivotal role in bridging the gap between research and practice. Clinical nurses play a dual role as both recipients and transmitters of evidence. Dissemination not only enables nurses to more easily access, understand, and apply the best available evidence in real-world settings but also allows them to share the evidence they have engaged with among their peers. Effective dissemination strategies can accelerate the adoption of research findings, enhance the accessibility and usability of evidence, and promote equity in evidence-based care delivery [7]. Therefore, strengthening evidence dissemination is fundamental to the successful implementation of evidence-based nursing practice.

While various strategies from printed material to social media have been developed [8], current evidence dissemination methods remain inadequate in addressing the needs of health care professionals. First, frontline nurses often encounter difficulties in accessing up-to-date, high-quality evidence, especially when facing language barriers and a lack of database resources [9]. Second, existing dissemination pathways are predominantly static and text-heavy, lacking structured formats and efficient retrieval mechanisms that support clinical decision-making [10]. Additionally, the lack of interactivity and intelligent recommendation systems limits personalized evidence delivery. Delays in updating digital platforms further hinder timely access to emerging research findings [11]. Therefore, developing a scientific and efficient evidence dissemination method remains essential to facilitate the transition of evidence from static dissemination to dynamic interaction.

In recent years, large language models (LLMs) have emerged as a pivotal research focus in the field of artificial intelligence (AI), offering substantial potential to revolutionize evidence dissemination in health care. By leveraging advanced natural language processing capabilities, LLMs can assist health care professionals in shifting from static internet-based information searches to dynamic, AI-driven knowledge acquisition [12]. In clinical nursing settings, LLMs are increasingly applied to a range of tasks, including supporting and optimizing clinical decision-making, generating nursing care plans, and providing medical inquiries and intelligent question-answering [13]. LLMs represent a promising new pathway to evidence dissemination in health care. They offer significant potential to enhance human-computer interaction and improve efficiency, facilitating the integration of evidence into routine clinical workflows and helping to bridge the gap between research and practice. Their scalability and adaptability make them valuable for expanding access to high-quality evidence, particularly for nurses with limited support in evidence retrieval.

However, existing general-purpose LLMs such as ChatGPT and Gemini lack domain-specific knowledge, exhibit poor alignment with users’ actual needs, and provide responses with relatively low accuracy [14]. Given the low fault tolerance in the health care field, developing specialized LLMs tailored to the medical domain to enhance accuracy is essential. Although an increasing number of studies have focused on developing medical LLMs by integrating medical knowledge into model training, most of these models are designed primarily for physicians and may not adequately address the distinct needs of clinical nurses. Therefore, developing LLMs tailored to nursing practice is essential to facilitate the dissemination of evidence-based nursing knowledge to nurses and ultimately improve patient care outcomes.

We aim to develop a highly interactive, responsive, and accurate system based on LLMs to support the dissemination of nursing evidence. To ensure its usability, it is essential to first understand the barriers that nurses encounter in the process of evidence dissemination, as well as their expectations for how LLMs can support and enhance this process. The aim of this study is to identify the challenges and barriers clinical nurses face in disseminating evidence, to examine their perspectives on the advantages and limitations of using existing LLMs for evidence dissemination, and to explore their needs and preferences regarding an LLM-based nursing evidence question-answering system. Focus group discussions and the nominal group technique (NGT) were used to gather clinical nurses’ perspectives on the content and functionality of the system, thereby informing its development.

## Methods

### Overview and Study Design

The development of the system’s content and function was determined using a combined focus group and NGT, which merged the in-depth discussion of a focus group with the prioritization process of a standard NGT. The focus group was conducted to identify the challenges and barriers that clinical nurses face in disseminating nursing evidence, as well as to examine their perspectives on the advantages and limitations of using existing LLMs for evidence dissemination. Building on these insights, the NGT was then used to further explore their needs and preferences regarding the LLM-based system to be developed. Both sessions were held online to overcome geographical constraints and busy schedules. The whole study was conducted between November 2024 and April 2025.

### Sampling and Recruitment

The study participants were clinical nurses recruited from the evidence application bases affiliated with the Fudan University Centre for Evidence-Based Nursing, all of whom had participated in clinical evidence application projects. The inclusion criteria for participants were as follows: (1) formal registered nurses who worked in the evidence application bases of the Fudan University Centre for Evidence-Based Nursing, (2) have participated in a completed clinical evidence application project, (3) have experience with and knowledge of LLMs, and (4) willingness to participate in the interview. The exclusion criterion was participants who withdrew from the research midway.

A purposive sampling method was used to select nurses with diverse specialties, professional titles, and years of experience to participate in focus group interviews and nominal group. Researchers publicly recruited eligible clinical nurses via social media (WeChat; Tencent), posting study information in multiple WeChat groups that collectively included over 500 registered nurses who had previously been trained in clinical evidence application by the Fudan University Centre for Evidence-Based Nursing, and informed them of the study’s purpose, methods, and significance to gauge their interest and willingness to participate. Those who expressed consent were invited to provide their contact information and their use of LLMs, enabling the researchers to screen participants and subsequently coordinate the dates and times of the focus groups. Researchers contacted the target participants and obtained informed consent. Data collection commenced only after a thorough informed consent process had been completed.

### Phase A: Focus Group

#### Data Collection

Clinical nurses completed a brief demographic survey and participated in 2 focus groups, each lasting approximately 90 minutes. The interview outline (Table S1 in Multimedia Appendix 1) was self-designed based on the research objectives. We conducted 2 focus groups between November and December 2024. The 2 focus groups were conducted online via Tencent Meeting (VooV Meeting), with one group consisting of 10 participants and the other of 12 participants. All participants kept their cameras and microphones on, which facilitated smooth communication and encouraged active participation. Each focus group was led by 2 female researchers, with one (WX) leading the focus group and the other (JR) taking notes. Both researchers were trained in focus group facilitation and had professional expertise in evidence-based nursing. They had no personal or professional conflicts of interest related to the study and maintained neutrality throughout the discussions. No prior relationship existed between the researchers and participants. Both audio and video recordings of the sessions were captured using the built-in recording feature of Tencent Meeting. Each focus group followed a four-step procedure: (1) participant and facilitator self-introductions; (2) an overview of the study background, concepts, and objectives; (3) guided discussion based on a semistructured interview guide, with balanced participation and thorough exploration of each topic; and (4) a brief summary and conclusion of the session.

Transcripts derived from Tencent Meeting’s live transcript function were reviewed by 2 team members (JR and YT) for accuracy. Data saturation was considered reached after 2 focus groups. Given the diversity of participants and the depth of discussions, the research team carefully reviewed the transcripts and coding, confirming that the major themes relevant to the study objectives were consistently captured. Therefore, we judged that additional focus groups were unlikely to yield substantially new information.

#### Data Analysis

After the interviews, the researchers imported transcripts into NVivo (version 14; Lumivero) for data storage and management. The transcripts were analyzed by using qualitative content analysis, based on the approach of Graneheim and Lundman [15]. The content analysis was performed by 2 researchers (JR and YT). The researchers independently reviewed the verbatim transcriptions to gain a thorough understanding of the content. Meaningful statements were identified and coded, with similar codes grouped into subcategories. Through an iterative process of abstraction, the subcategories were further organized into broader categories and ultimately consolidated into overarching themes. Representative participant quotations were identified to illustrate each theme. Any disagreements were discussed, and if no agreement could be reached, the third researcher (WX) helped resolve the disagreement. Additionally, the research team held regular meetings to review and refine the codes and themes, ensuring a consistent and rigorous analytical process.

### Phase B: Nominal Group

#### Overview

The NGT is a qualitative exploratory research method that can be used for problem-solving, decision-making, priority setting, and achieving consensus on complex issues [16]. NGT allows individuals to generate ideas independently without the influence of others, offering advantages such as time efficiency, reduced bias, and the promotion of equal participation among all members.

We conducted the nominal group 2 months after the focus groups. The NGT followed a 5-stage process [17]: (1) introducing and presenting the nominal question, (2) generating ideas independently, (3) sharing ideas in a round-robin format, (4) clarifying and discussing the ideas collectively, and (5) independently voting and ranking the ideas to establish priorities. To overcome geographical constraints and busy schedules, this part of the study was conducted asynchronously using electronic questionnaires and social media. We adapted the NGT with reference to a previous study [18]. The specific steps were as follows.

#### Silent Idea Generation

Participants were invited to independently reflect on the nominal question: “If developing a large language model–based nursing evidence question-answering system, what functions would you hope it includes?” This question constituted the silent idea generation stage of the NGT. The focus group results were also shared with the participants, allowing them to reflect on their functional needs for the system to be developed in light of the identified barriers to evidence dissemination. To provide sufficient time for thoughtful consideration and to reduce the influence of others’ opinions, individual responses were collected via an online questionnaire.

#### Sharing Ideas

The researcher collated ideas and made them publicly available to participants. Participants were able to review and reflect on each idea prior to the formal discussion, and were encouraged to note any unclear ideas in advance to be well-prepared for the discussion.

#### Clarification and Discussion

The clarification and discussion phase was facilitated by the researchers through an online discussion within the WeChat group, allowing participants to ask questions and clarify the meaning of the ideas. This ensured that all members fully understood the summarized functions of the LLM-based evidence question-answering system.

#### Voting and Ranking

In this step, 2 rounds of voting were organized. The first round aimed to identify the most critical system functions, and the second round focused on prioritizing these functions. In the first round of voting, participants were invited to rate each function on a scale from 1 to 9, with lower scores indicating less need for the function and higher scores indicating greater need. The researcher collated and analyzed the results by calculating the average score for each function and ranking them in descending order. The top 70% (9/13) of functions with average scores were retained, while those ranked lower were removed. The retained functions advanced to a second round of voting, during which participants were asked to rank the functions in order of importance to determine their priority. The researcher collected all participants’ rankings and converted them into scores, assigning a value of 1 to the highest-ranked function. The average ranking for each function was then calculated, with lower values indicating higher priority.

### Ethical Considerations

The Institutional Review Board of the School of Nursing, Fudan University, reviewed the study and determined that it did not require full ethical approval and involved minimal risk. Prior to the research, all participants provided informed consent and were informed that they could withdraw from the study at any time. To ensure anonymity, we used the code A to represent the first focus group and B for the second focus group, with numbers indicating individual participants. Only the researchers have access to the data. This study was conducted in compliance with the Declaration of Helsinki and was reported according to the items of the COREQ (Consolidated Criteria for Reporting Qualitative Research) checklist (Checklist 1 [19]). Participation was voluntary, and no material compensation was provided. As a benefit of their participation, participants were informed that, if the LLM for nurses developed by our research team were successfully completed in future studies, they would be granted priority access to its trial use.

## Results

### Participant Details

A total of 38 nurses responded to the study invitation. Following eligibility screening and obtaining consent, 22 participants were recruited, and none withdrew before the study’s completion. Participant demographics and characteristics are detailed in Table 1. The participants in the focus group and the nominal group were the same. The participants were experienced clinical nurses with a mean age of 37.2 (range 28-52) years, the majority of whom held a master’s degree (n=13). The participants’ professional designation was mainly concentrated at the levels of charge nurse and deputy director of nursing, with the majority serving as head nurses (n=11), followed by nursing administrators (n=5). Participants were primarily drawn from intensive care units (n=7), general wards (n=6), and nursing administration departments (n=5).

**Table 1:** Demographic and professional characteristics of the participants (N=22).

Characteristics			Values
Age (years), mean (range)			37.2 (28-52)
**Education level, n (%)**			
	Bachelor’s degree	6 (27)	
	Master’s degree	13 (59)	
	Doctoral degree	3 (14)	
**Professional designation, n (%)**			
	Senior Registered Nurse	3 (14)	
	Charge Nurse	9 (41)	
	Deputy Director of Nursing	9 (41)	
	Director of Nursing	1 (4)	
**Position, n (%)**			
	Nurses	3 (14)	
	Head nurse	11 (50)	
	Clinical teacher	3 (14)	
	Nursing administrator	5 (23)	
**Department, n (%)**			
	General wards	6 (27)	
	Intensive care units	7 (32)	
	Emergency departments	3 (14)	
	Operating rooms	1 (4)	
	Nursing administration departments	5 (23)	

### Focus Group Results

During the focus group, participants initially generated ideas by freely sharing their individual experiences and perspectives in response to the guiding questions. The facilitator guided other members to respond to and expand upon these viewpoints, while differing opinions were clarified and contrasted through interaction. By summarizing key points, the facilitator helped participants further articulate their perspectives and fostered deeper discussion. Over the course of these exchanges, individual viewpoints evolved into more structured collective insights, which were subsequently synthesized into the main themes and subthemes presented.

Each participant contributed their perspectives during the focus group. The analysis of the interviewees’ transcripts generated 157 codes. A total of 3 main themes and 7 subthemes emerged from the data analysis (Table 2). The three main themes identified were (1) pathways for evidence dissemination among nurses, (2) barriers that hinder the effective dissemination of evidence, and (3) advantages and limitations of using LLMs to support evidence dissemination. Participants discussed the barriers within existing evidence dissemination pathways and highlighted the advantages of using LLMs for evidence dissemination. Meanwhile, they recognized the limitations of current LLMs, which laid the foundation for their subsequent reflections in the nominal group discussions on the desired functionalities of a newly developed LLM.

**Table 2:** Themes and subthemes identified from focus group discussions with clinical nurses on barriers to evidence dissemination and large language model (LLM)–based support.

Main theme and subtheme		Codes
**Pathways for evidence dissemination among nurses**		
	Evidence dissemination pathways to nurses	Databases Platforms provided by professional associations Social media Materials and sessions organized by nursing administrators
	Nurses’ evidence dissemination pathways to peers	Organize training sessions, lectures, and academic exchange meetings Integrate the latest evidence into routine nursing practices Social media
**Barriers that hinder the effective dissemination of evidence**		
	Organizational and environmental barriers	Restricted access to databases Limited channels for evidence dissemination
	Nurse-related barriers	Lack of time and energy Low motivation Insufficient skills in systematic search of evidence
	Evidence-related barriers	Excessive and fragmented nature of available evidence Difficulties in understanding evidence
**Advantages and limitations of using LLMs to support evidence dissemination**		
	Advantages of LLMs in overcoming barriers to evidence dissemination	Rapid response Literature summarization Literature translation
	Limitations of LLMs hinder their widespread use in evidence dissemination	High demands on user input formulation Lack of domain-specific expertise in general-purpose models Insufficient scientific rigor

### Theme 1: Pathways for Evidence Dissemination Among Nurses

The evidence dissemination process involved the dissemination of evidence from original sources to nurses, who then further disseminated it to their peers. This theme highlighted the various pathways used for evidence dissemination among nurses, which were further categorized into 2 subthemes: evidence dissemination pathways to nurses and nurses’ evidence dissemination pathways to peers.

#### Evidence Dissemination Pathways to Nurses

To better understand how evidence is disseminated from its original sources to nurses as recipients, we focused our observations on how they access evidence. Nurses accessed evidence through proactive information-seeking or received it passively through managerial staff. When proactively accessing evidence, clinical nurses often rely on traditional yet more scientific methods, such as databases and platforms provided by professional associations.

For those of us who are more involved in research, we mainly access evidence from more traditional ways, such as databases or some professional websites. We are still using these methods now.

A12

When I encounter a problem, I first check UpToDate, because it is regularly updated and its content is reviewed by experts in the relevant fields. Therefore, it is a resource that aligns well with our clinical needs.

A10

When seeking relatively stable and widely recognized foundational knowledge that does not frequently change, they preferred innovative and fast-response pathways, including social media platforms like WeChat and Rednote, or AI tools.

Since WeChat is widely used nowadays, I often access evidence through official accounts. I also follow various medical journals, and some experts and professors share expert consensus statements or guidelines in their WeChat Moments or group chats.

B4

Whether I am relaxing or working, I can easily open apps—it is convenient and motivates me to use it. Most of the time, for quick access to basic evidence, I rely on some mobile apps, including AI tools.

A3

The participants also reported that they sometimes received evidence passively through a top-down pathway. For example, nursing administrators would distribute materials and organize sessions for nurses to learn about the latest evidence.

Sometimes, evidence is disseminated through training sessions organized by professional associations. They issue certain industry standards to be implemented in clinical settings, organize us to study them, and we are then requested to follow these standards in practice.

B7

#### Nurses’ Evidence Dissemination Pathways to Peers

After accessing the evidence, clinical nurses had the responsibility to further disseminate it to their peers. In disseminating evidence to peers, nurses in different roles adopted distinct pathways. For nursing administrators, they could leverage their positional authority to organize training sessions, lectures, and academic exchange meetings, or integrate the latest evidence into routine nursing practices.

I usually disseminate evidence through sessions, presenting the evidence to nurses during their professional training sessions or morning meetings.

B1

During the revision of nursing routines, we prioritize incorporating evidence that we have generated ourselves and that has proven to be effective in practice, thereby facilitating its promotion throughout the entire hospital.

B10

Additionally, most nurses used social media to disseminate evidence to their peers.

My personal favorite method of dissemination is through WeChat—sharing links from official accounts in group chats, or sending good articles and guidelines I come across while searching the literature.

B4

### Theme 2: Barriers That Hinder the Dissemination of Evidence

Although various pathways were used to disseminate evidence, there were still some barriers that hindered these processes. These barriers could be categorized into 3 main aspects: organizational and environmental barriers, nurse-related barriers, and evidence-related barriers.

#### Organizational and Environmental Barriers

Organizational and environmental barriers included restricted access to databases and limited channels for evidence dissemination. Some participants indicated that their hospitals had not subscribed to certain databases, limiting their access to evidence resources. Such restricted access might lead to missed opportunities to access valuable and up-to-date information.

When we provide guidance to other regions that are not affiliated with universities, they often face difficulties in accessing data. They may not have permission to use certain databases when searching for information, or they may need to pay to download articles.

A12

Although some nurses ultimately accessed the evidence they needed, the process often demanded significant effort, including drawing on extensive personal networks or incurring financial expenses.

Some of the data was inaccessible to me, so I had to rely on my own resources—often asking my family members to help download information that I couldn’t access locally, even though it might be easily accessible at other hospitals.

A4

Limited resource sharing within and between hospitals hindered effective evidence use. Nurses who had access to the latest evidence often lacked the channels and resources to disseminate it, while those from other departments or hospitals were eager to access such resources to reduce their evidence-gathering burden.

The evidence I retrieved also had issues with dissemination. For example, we recently admitted a patient with Candida auris, and I found information on the appropriate concentration of an antifungal solution. However, I was not sure how to disseminate this information—should I report it to the hospital?

A10

I was thinking whether hospitals could establish a shared platform. Some evidence can be found in published literature, but in most cases, it’s unpublished. Is there a way we can share such resources and learn from each other?

B2

#### Nurse-Related Barriers

Lack of time and energy, low motivation, and insufficient skills in systematically searching for evidence were barriers to evidence dissemination among clinical nurses. Due to the heavy workload, clinical nurses often lacked the time and energy to search for evidence. Evidence summaries, similar to guidelines, were the most common form of evidence dissemination, offering more systematic and comprehensive evidence. However, producing such summaries was both labor-intensive and time-consuming, which hindered nurses from promptly accessing evidence and efficiently disseminating it to their peers.

Actually, I still prefer using traditional methods—searching, screening, and appraising evidence—to ultimately produce a summary. However, the process takes a relatively long time to complete.

A2

Even after accessing the literature, identifying relevant evidence might still impose a reading burden on clinical nurses. When the literature was not in their native language, language barriers further exacerbated this burden.

Sometimes, although there are guidelines or expert consensus documents available, to be honest, there is not much time to thoroughly read through the entire content.

B4

Additionally, nurses’ limited skills in systematic searching further hindered the process of evidence dissemination from original sources to nurses, as many general nurses were not proficient in retrieving evidence through databases.

I learned how to conduct systematic searches by following experts, but I found that some of the other nurses in clinical practice hadn’t received such training. They often felt lost and didn’t know how to proceed.

B8

On the other hand, some clinical nurses lacked motivation to actively seek out evidence. Even when they were exposed to it passively through meetings or training sessions, they often did not pay close attention and were likely to forget the latest evidence shortly afterward.

I felt that many nurses weren’t very interested or attentive. I think they probably didn’t remember the information clearly at the time and later came back to ask about it again…So the biggest challenge I faced in disseminating evidence was their low engagement and how to help them remember the evidence better.

B1

#### Evidence-Related Barriers

Evidence-related barriers included the excessive and fragmented nature of available evidence and difficulties in understanding evidence. Participants reported that the current evidence was excessive and fragmented, making it challenging for clinical nurses to identify and apply it effectively in practice.

Now, there is an overwhelming amount of evidence. With the increase in sources, the content has also become more diverse, which makes clinical practice more confusing. In this era of evidence overload, implementing evidence in clinical settings has actually become more challenging.

A11

Participants also reported difficulties in understanding evidence. Given the complexity of real-world clinical settings, some evidence was seen as overly simplistic and lacking practical guidance. In addition, some evidence lacked proper interpretation, making it difficult for nurses to fully understand and apply it, even when it was available.

Our current challenge is that the group standards are very simple, and there is a lack of interpretation when using them. No one is effectively explaining the evidence behind each item and in what context it should be applied.

B9

Even when the evidence is fully presented in front of me, applying it in clinical practice can still be challenging. How should I interpret it? What does the evidence actually mean? What actions align with its recommendations? Without someone to explain it, many difficulties arise.

B7

### Theme 3: Advantages and Limitations of Using LLMs to Support Evidence Dissemination

The recent development of LLMs has introduced new pathways to evidence dissemination. This theme focused on the advantages and limitations of using LLMs to support evidence dissemination, which were further categorized into 2 subthemes: advantages of LLMs in overcoming barriers to evidence dissemination and limitations of LLMs that hinder their widespread use in evidence dissemination.

#### Advantages of LLMs in Overcoming Barriers to Evidence Dissemination

The advantages of LLMs in rapid response, literature summarization, and translation could help overcome some of the existing barriers to evidence dissemination. The question-answering chat format of LLMs enabled nurses to quickly access answers, reducing the difficulty of gathering evidence from scratch and saving time.

When I encounter something unfamiliar or have questions, I can quickly look it up using an LLM, and I find it quite convenient to use.

B3

LLMs possessed powerful literature summarization capabilities. Users could submit individual articles for key information extraction. Furthermore, LLMs could also effectively organize and synthesize content from multiple sources. This function consolidated excessive and scattered evidence, significantly reducing the burden of reading large volumes of original materials.

I once used AI for this purpose: I wanted to understand the development of Enhanced Recovery (ERAS) in our country. So, I searched for it, and the AI clearly laid out the entire timeline, key viewpoints, and relevant documents.

B6

Additionally, the translation capabilities of LLMs assisted in overcoming language barriers, lessening the reliance on nurses’ language proficiency, and facilitating the reading and understanding of evidence written in nonnative languages.

AI has translation capabilities. If you don’t understand something, it can help you translate it. We can leverage this function to assist with translating articles written in languages other than English and Chinese.

A8

#### Limitations of LLMs Hinder Its Widespread Use in Evidence Dissemination

The limitations of LLMs included their high demands on user input formulation, lack of domain-specific expertise in general-purpose models, and insufficient scientific rigor. Some participants revealed that the quality of LLM-generated responses was closely related to the quality of user input, noting that current LLMs place high demands on how users formulate their questions.

AI places high demands on the instructions you give it—if your prompt is well-crafted, it can provide a relatively good response. However, for someone like me who lacks experience, it can actually be quite a challenge.

B1

Currently, clinical nurses commonly use general-purpose LLMs and report that these models lack sufficient medical expertise and demonstrate poor understanding and application of specialized terminology.

The language used by AI is still often imprecise, especially in professional fields. For example, I work in critical and emergency care, and AI shows a significant lack of specialized vocabulary in this area.

A11

Participants also found that LLM-generated content could be deceptive, often fabricating answers and sources, with responses lacking scientific rigor. Due to insufficient scientific rigor, nurses presented little trust in LLM-generated content.

I’ve found that sometimes it gives answers that sound perfectly reasonable and convincing, but in reality, they are completely made up. When you verify them in the databases, you find that there’s no supporting evidence at all.

A5

When it comes to accessing evidence, we don’t quite trust AI because its responses lack sufficient support from the literature and are therefore considered unreliable.

A9

#### Nominal Group Results

Building on the findings from the focus group discussions, an NGT session was conducted to further refine the expected LLM functionalities and determine their priorities. Table 3 shows the complete results of the NGT session, including 13 subfunctions and the results of the 2 rounds of voting.

Ideas were generated independently and tabulated. A total of 4 function categories (question-answering, text rewriting, evidence management and sharing, and feedback) and 13 subfunctions emerged from the NGT session. To facilitate participants’ understanding of the functions, the researchers provided a description next to each subfunction (Table S2 in Multimedia Appendix 1).

During the clarification and discussion phase, all participants indicated that they understood the proposed 13 functions and had no additional suggestions or modifications.

Voting was conducted in 2 rounds, each achieving a 100% (22/22) response rate. The first round of voting was conducted on the 13 subfunctions. The top 70% (9/13) of functions with average scores were retained for further voting. Ultimately, 9 functions were retained and 4 were removed. Participants were presented with the retained and removed functions. The second round of voting was conducted on the 9 subfunctions to determine their relative priorities. The top 3 ranked functions were evidence-based, high-quality question-answering, evidence source provision, and personalized evidence recommendation.

**Table 3:** Ranking of candidate functions for a large language model–based nursing evidence question-answering system using the nominal group technique.

Number	Subfunctions	First round		Second round		
		Score, mean (SD)	Decision	Rank score, mean (SD)	Final ranking	
1	Evidence-based high-quality Q&A^a^	9.00 (0.00)	Retain	1.23 (0.69)	1	
2	Evidence source provision	8.68 (0.57)	Retain	3.09 (1.80)	2	
3	Personalized evidence recommendation	8.45 (0.80)	Retain	4.64 (2.22)	3	
4	Evidence update push notification	8.23 (0.97)	Retain	5.27 (2.39)	5	
5	Summarization	8.18 (1.01)	Retain	5.05 (1.81)	4	
6	Question prompt assistance	8.14 (0.99)	Retain	5.59 (1.89)	6	
7	User sharing	8.09 (0.97)	Retain	6.59 (2.11)	7	
8	Patient material readability optimization	8.00 (0.98)	Retain	6.73 (2.00)	8	
9	User feedback channel	8.00 (1.15)	Retain	6.82 (2.15)	9	
10	Evidence application case display	7.91 (1.44)	Remove	N/A^b^	N/A	
11	Text comparison	7.77 (1.15)	Remove	N/A	N/A	
12	Literature translation	7.45 (1.44)	Remove	N/A	N/A	
13	Language optimization	7.18 (1.44)	Remove	N/A	N/A	

^a^Q&A: question-answering.

^b^N/A: not applicable.

## Discussion

### Principal Findings

This paper presented the findings from a qualitative study using focus group and NGTs. Focus group was conducted to identify the challenges and barriers that clinical nurses face in disseminating evidence, as well as to examine their perspectives on the use of existing LLMs to support evidence dissemination. Then, the NGT was conducted to explore nurses’ needs and preferences for the system to be developed. The focus group results yielded 3 main themes and 7 subthemes. The 3 main themes identified were pathways for evidence dissemination among nurses, barriers that hinder the effective dissemination of evidence, and advantages and limitations of using LLMs to support evidence dissemination. The NGT sessions ultimately identified 9 desired functions. After prioritization, the top 3 ranked functions were evidence-based, high-quality question-answering, evidence source provision, and personalized evidence recommendation. The results from both the focus group and NGT sessions served to guide the subsequent development of the system.

Our study found that the process of evidence dissemination, which involves the transfer of evidence from original sources to nurses and subsequently from nurses to their peers, occurs through various pathways, including training sessions, conferences, databases, online platforms, and social media. The findings reported in a systematic review were also reflected in our findings [8]. With the rapid advancement of AI, LLMs are increasingly being used to provide medical information to health care providers. In this context, a previous study found that health care professionals viewed ChatGPT as valuable for accessing the latest research and evidence-based guidelines, supporting diagnosis and treatment decisions, answering clinical questions, summarizing articles, and generating patient education materials [20]. However, the content generated by general-purpose LLMs must be critically evaluated before being considered reliable evidence, as its accuracy and sources may be uncertain. When the generated content is explicitly based on high-quality, verifiable sources, such as clinical guidelines or systematic reviews, it can be regarded as evidence, while users should simultaneously remain cautious. Regardless, the emergence of LLMs has introduced a new pathway for evidence dissemination.

While barriers to evidence dissemination exist across the broader health care field, clinical nurses have relatively limited opportunities for advanced education and specialized training in evidence-based knowledge, making the challenges they face in evidence dissemination particularly pronounced [2]. Prior to the advent of LLMs, evidence dissemination faced significant barriers, including those stemming from the organizational and environmental factors, the nurses themselves, and the nature of the evidence itself, further limiting the adoption of evidence-based practice. Among these barriers, the most prominent issues were the lack of time and energy, limited access to databases, and the excessive and fragmented nature of available evidence. Adequate support resources were urgently needed to enhance the dissemination of evidence among nurses. Consistent with our findings, a study by Halili et al [21] suggested that abundant and accessible resources promoted evidence dissemination by supporting the retrieval, integration, and implementation of evidence in clinical settings, enabling clinical nurses to convey the latest evidence to health care professionals, patients, and their families. In addition, a study showed that information overload caused significant information anxiety among nurses, which negatively impacted their core competencies and hindered the adoption of evidence-based practice and clinical decision-making [22]. Addressing these barriers is essential for advancing evidence-based practice.

The advantages of LLMs offered promising solutions to the challenges of evidence dissemination. Our study suggested that LLMs could serve as a valuable source of evidence. Their rapid question-answering and literature summarization functions helped clinical nurses efficiently access evidence, overcoming previous limitations of resource scarcity, while their language proficiency enhanced nurses’ understanding of the evidence. A study also highlighted that LLMs offer unprecedented potential to rapidly disseminate critical health information and transcend linguistic barriers, aligning with our findings [23].

Although LLMs can accelerate the evidence dissemination process, there are still several limitations that lead to a suboptimal user experience for nurses. A particularly prominent limitation is the lack of transparency and explainability in LLM-generated content. Cacciamani [24] highlighted a similar concern, noting that LLM-delivered information often lacked transparency regarding its sources, leaving users unable to verify or control the origin of the content. Due to the lack of evidence support in LLM-generated answers and the occasional generation of incorrect information, nurses tend to mistrust them. Additionally, our participants noted that LLMs demand precise and well-formulated inputs, which they lacked the skills to provide. A cross-sectional qualitative study reported low adoption of LLM AI tools among Chinese hospital administrators, citing mistrust in accuracy and limited prompting skills as key barriers to wider use [25]. To better integrate LLMs into health care field, further improvements to the models are necessary.

Through NGT sessions, our research found that clinical nurses have put forward a range of functional requirements for LLM tools designed to enhance evidence dissemination. The most critical functional needs were evidence-based, high-quality question-answering, provision of evidence sources, and personalized evidence recommendations. A study demonstrated that low risk perception and high perceived ease of use foster greater trust, ultimately increasing users’ intention to adopt the system [26]. We believed that the provision of evidence-based answers with clearly cited sources could increase clinical nurses’ trust in and intention to use an LLM-based system. Additionally, personalized evidence recommendations could tailor content based on specific clinical roles or patient populations, addressing the variability in evidence needs across different nursing contexts.

In light of the aforementioned limitations and needs, some studies have already begun making efforts to optimize LLMs. At the technical level, multiple approaches have been used to improve the accuracy of LLMs, such as prompt engineering, retrieval-augmented generation, and fine-tuning [27]. Other researchers [28] also recognized the importance of providing evidence sources and developed an LLM based on medical guidelines that not only generated answers but also provided source citations. These functional requirements informed the development of LLMs specifically tailored to clinical nursing practice. To ensure LLMs become reliable and trustworthy tools in evidence-based nursing practice, future system development should prioritize not only technical performance but also user needs, usability, and integration within the clinical nursing environment to enhance adoption and impact.

### Limitations

This research has some limitations that need to be acknowledged. First, both the focus group and nominal group sessions were conducted online. While virtual meetings allowed for broader participation by overcoming geographical and scheduling constraints, they may not be as rich in interaction as face-to-face sessions, potentially limiting the depth and dynamics of group discussions. Second, the majority of participants in our study were clinical nurses with higher educational backgrounds or senior professional designations. As a result, the findings may not fully capture the views of frontline or less experienced nurses, who may have different experiences, needs, and challenges related to evidence dissemination and the use of LLMs. Their perspectives might reveal additional functional requirements or usability concerns that were not captured in this study. Therefore, future research could include a wider range of nurses to ensure the system meets the needs of diverse clinical users.

### Conclusions

The current process of evidence dissemination is hindered by multiple barriers, including organizational and environmental barriers, nurse-related barriers, and evidence-related barriers. LLMs hold significant promise as innovative tools for supporting evidence dissemination, with the potential to overcome current barriers and enhance the implementation of evidence-based nursing practice. However, further refinement and optimization are necessary to fully realize their potential. Clinical nurses have put forward a range of functional requirements for LLM tools designed to enhance evidence dissemination, thereby informing the development of LLMs specifically tailored to clinical nursing practice.

## Appendix

Multimedia Appendix 1 Focus group interview outline and candidate functions of a large language model–based nursing evidence question-answering system.

## Abbreviations

AI: artificial intelligence
COREQ: Consolidated criteria for Reporting Qualitative research
LLM: large language model
NGT: nominal group technique

## Data Availability

The datasets generated or analyzed during this study are available from the corresponding author upon reasonable request.
