# Supplementary material for: Nurses’ Perspectives on Evidence Dissemination Barriers and Large Language Model–Based Support: Qualitative Study Using Focus Groups and Nominal Group Technique
Source: J Med Internet Res. 2025 Nov 7;27:e80289. doi: 10.2196/80289 (PMC12639338; doi:10.2196/80289)
Supplement: Multimedia Appendix 1 [file jmir_v27i1e80289_app1.docx]

**Table S1.** Focus group interview outline

| Interview questions |
| --- |
| 1. What challenges have you encountered in various stages of accessing evidence? |
| 1. What difficulties have you experienced in disseminating evidence to others? |
| 1. What role do you think artificial intelligence, particularly large language models, can play in these processes? What are their advantages and limitations？ |

**Table S2.** Candidate functions of a large language model–based nursing evidence question-answering system

| Function category | Sub-function | Description |
| --- | --- | --- |
| Question-answering | Evidence-based high-quality Q&A | Provide fast, precise, and comprehensive answers supported by high-quality evidence. |
|  | Personalized evidence recommendation | Recommend tailored evidence based on the user's submitted patient cases. |
|  | Evidence source provision | Provide original sources such as literature and web links for evidence traceability. |
|  | Question prompt assistance | Provide related or follow-up questions based on vague queries to assist the user in refining their inquiries. |
| Text rewriting | Text comparison | Compare differences between multiple uploaded texts, e.g., differences across versions of clinical guidelines. |
|  | Summarization | Extract and summarize content from uploaded materials. |
|  | Literature translation | Translate literature from other languages into the user's native language. |
|  | Language optimization | Polish and refine existing text materials. |
|  | Patient material readability optimization | Generate patient-friendly educational materials from professional documents like clinical evidence. |
| Evidence management and sharing | Evidence updates push notification | Push updated evidence dynamically when previously consulted evidence is revised. |
|  | User sharing | Allow users to upload materials to share nursing protocols, workflows, clinical procedures, health education materials, and psychological cases. |
|  | Evidence application case display | Demonstrate real-world implementation, methodology, supporting data, and usage scenarios from domestic and international cases. |
| Feedback | User feedback channel | Enable users to provide feedback or dissatisfaction with ai responses, facilitating model learning and improvement. |
